# Calculating the economic burden of presumed microbial keratitis admissions at a tertiary referral centre in the UK

**DOI:** 10.1038/s41433-020-01333-9

**Published:** 2020-12-07

**Authors:** George Moussa, James Hodson, Nick Gooch, Jasvir Virdee, Cristina Penaloza, Jesse Kigozi, Saaeha Rauz

**Affiliations:** 1 Birmingham and Midland Eye Centre, Sandwell and West Birmingham Hospitals NHS Trust, Birmingham, UK; 2 Academic Unit of Ophthalmology, Institute of Inflammation and Ageing, University of Birmingham, Birmingham, UK; 3 Department of Biostatistics, Institute of Translational Medicine, University Hospitals Birmingham NHS Foundation Trust, Birmingham, UK; 4 Health Economics Unit, Institute of Applied Health Research, University of Birmingham, Birmingham, UK

**Keywords:** Health care economics, Business and industry, Corneal diseases

## Abstract

**Purpose:**

Microbial keratitis (MK) is the most common non-surgical ophthalmic emergency admission in the UK. However, few prospective health-economic studies of MK have been performed, and no specific healthcare resources group (HRG) code exists. This study is designed to determine the feasibility of a data collection tool derived from the microbiology ophthalmology group (MOG) clinical record form, to enable quantification of direct costs of inpatient care, as well as prospective capture of epidemiological data relating to outcomes of MK.

**Methods:**

Clinical, demographic and economic data were collected retrospectively between January and December 2013 for 101 consecutive patients admitted with MK, using an adaption of the MOG toolset. The direct cost of admission (COA) was calculated using national reference costs and compared to actual income to generate profit/deficit profiles for individual patients. Indices of multiple deprivation were used to assess effect of deprivation on the COA.

**Results:**

The total income generated through discharge coding was £252,116, compared to a COA of £357,075, yielding a deficit of £104,960 (median: £754 per patient). The cost deficit increased significantly with length of stay (LOS, *p* < 0.001), whilst patients with short LOS were income generators; cost neutrality occurred at 4.8 days. Greater socioeconomic deprivation was also associated with a significantly higher cost deficit.

**Conclusion:**

LOS is the key driver for COA of care for MK admissions. Protocols should encourage discharge of patients who are able to self-administer treatment after the sterilisation phase. The MOG-derived data collection toolset captures pertinent clinical data for quantification of COA. Further development into a multiuser and multisite platform is required for robust prospective testing, together with expansion to capture indirect costs of disease burden, including impact of treatment, visual morbidity and quality of life.

## Introduction

Microbial keratitis (MK) is a leading cause of global blindness. Incidence, prevalence and underlying causative organisms vary across geographical regions [1], ranging from 11 per 100,000 in the USA to 799 per 100,000 per year in Nepal [2]. MK forms the most common non-surgical ophthalmic emergency in the UK, with an estimated incidence of 40–52 people per 100,000 per year [1]. Patients present to the emergency department with painful, reduced vision. Corneal scrapes for microscopy, culture and sensitivity and polymerase chain reaction (PCR) for pathogen DNA provide mainstay investigations. However, as corneal scrapes to microbiologically confirm a causative microbe are reported to have a sensitivity of only 23.7–61.5% [3], most reports of MK refer to a clinical diagnosis of presumed MK.

Management of MK involves initiating intensive antimicrobial therapy to sterilise the infection, with the chosen antimicrobial agent from extrapolation of likely organisms and culture sensitivities, derived from local institutional or regional microbiology datasets. This is then followed by strategies to promote re-epithelialisation and reduce the level of inflammation. Topical eye drop therapy is initiated hourly, day and night, with patients frequently asked to self-administer at home. However, treatment regimens are arduous, impacting upon compliance, quality of life and clinical outcomes. An estimated 36% of patients taking eye drops are non-compliant [4]; and this is frequently overcome by hospitalisation for a minimum of 24 h, although this is variable, and a longer duration is generally required.

The UK National Health Service (NHS) is a healthcare system that gathers data of clinical activity through coding. NHS Digital (previously Health and Social Care Information Centre) estimate 2617 admissions annually [5] for MK, but these data are limited, due to the absence of a healthcare resources group (HRG) code specifically for admitting a patient with MK for intensive therapy. Instead, patients are coded for diagnostic or operative procedures, including corneal glue (C474), superficial keratectomy (C451) and biopsy lesion of cornea (C511). Additional data collected including diagnoses, procedures, medication and length of stay (LOS) are translated at discharge by the hospital contracts department into HRG codes, according to a database published annually by the Department of Health (DoH) [6]. The overall cost assigned to this HRG code is then charged either to the Clinical Commissioning Group where the patient is registered or directly to NHS England (NHSE) if the treatment is categorised as a specialist procedure. Since these HRG codes are the determinants of income, gathering of inaccurate data that does not reflect the intensity or duration of treatment will contribute to losses in equity.

For MK admissions, LOS is a pivotal resource quenching factor. Payment from the commissioners is a fixed amount, independent of the actual LOS, as HRG codes have predefined payment and trim points that vary depending on diagnosis. Trim points denote the time point in days, during a patient’s admission, after which a hospital earns additional extra bed-day payments, which generate additional income for providing routine patient care for extended periods. This encourages rapid discharge, as there is no additional revenue generated for increased LOS up until the trim point. For example, “minor cornea or scleral procedure” has a trim point of 5 days, meaning that the hospital receives the same income for patients that have a LOS between 1 and 5 days. As such, earlier patient discharge would result in a lower cost of treatment, but no change in income, allowing for the potential to generate a profit. This encourages managerial strive to improve efficiency, minimise costs and discharge patients as early as is feasible, ideally without compromising patient care.

This study is designed to determine the feasibility of a data collection tool derived from the microbiology ophthalmology group (MOG) clinical record form [7]. Specific modifications of the MOG tool potentially enable (1) prospective epidemiological data capture relating to the management and outcomes of MK, (2) quantification of direct costs of inpatient care for MK, compared against income generated through hospital coding and tariffs and (3) the effect of socioeconomic deprivation of our cohort on our calculated costs. The tool was tested against a retrospective cohort. The ultimate aim is to develop and implement a multiuser platform with multisite access, which has the capability to robustly capture prospective patient cohorts in a national registry, ensuring a standardised dataset that highlights evolving microbial trends (phenotype, antimicrobial sensitivity, resistance), changing patient factors and refines the collection of direct and, importantly, indirect costs of care.

## Methods

### Data collection

Parameters and fields from the MOG data collection form (Supplementary File 1) were itemised and constructed in an Excel^®^ (Microsoft Corporation, Redmond, WA) spreadsheet format using four main domains: demographics, clinical features, microbiology and treatment (Supplementary File 2). The form was then used to collect data for a retrospective cohort of 101 consecutive patients admitted to the Birmingham and Midland Eye Centre (Birmingham, UK, a tertiary referral centre) between January and December 2013. Identification of patients was through two hospital data resources: (1) the ophthalmic unit ward MK logbook and (2) a MK admissions logbook, maintained prospectively by the ophthalmology responsible standing officer.

### Disease severity

Patients were stratified according to disease severity, as defined by Keay et al. [8]. Briefly, this comprises of a four step severity spectrum, as described below:

1. Grade 1: mild disease, accompanied by lesions of <4 mm in diameter outside the central 4 mm zone.
2. Grade 2: severe MK without visual loss, accompanied by culture negative or not cultured scrapes, with either any part of the lesion within the central 4 mm zone, or outside the central zone with hypopyon, or outside the central with a diameter ≥2 mm.
3. Grade 3: severe MK without visual loss, accompanied by culture positive scrapes.
4. Grade 4: severe MK with visual loss of ≥2 lines of best corrected visual acuity and/or surgical intervention.

Ocular risk factors were classified into contact lens wear, ocular surface disease (including dry eyes, cicatrising conjunctivitis, atopy), previous past ocular surgery and trauma, diabetes mellitus, topical and systemic immunosuppression, trauma and incomplete lid closure (from proptosis and/or exposure keratopathy from whatever cause).

### Socioeconomic deprivation ranking

Data are readily available describing multiple deprivation metrics in the UK. The English indices of deprivation 2019 are a unique measure of relative deprivation across England, calculated from seven different facets of deprivation, to which different weightings are applied:

1. Income deprivation (22.5%).
2. Employment deprivation (22.5%).
3. Education, skills and training deprivation (13.5%).
4. Health deprivation and disability (13.5%).
5. Crime (9.3%).
6. Barriers to housing and services (9.3%).
7. Living environment deprivation (9.3%).

These are combined to produce an overall weighted relative measure of deprivation, the Index of Multiple Deprivation (IMD) [9] for every postcode area in England. Based upon these ranks, deciles are then calculated, with areas in IMD decile 1 being the most deprived, and IMD decile 10 being the least deprived. The distribution of IMD deciles in the area around the Birmingham and Midland Eye Centre is visualised in the map in Supplementary Fig. 1.

Residential postcode data were recorded for all patients: this provided the input data enabled IMD decile assignment for each patient. These were then dichotomised for analyses, with deciles 1–5 classified as “more deprived” and deciles 6–10 as “less deprived”.

### Direct-cost analyses

Direct costs of admission (COAs) were calculated for individual patients; the components of the overall COA, and the sources of information used for costing are described below.

#### Bed days

The total cost per night for the use of a hospital bed, excluding staffing or treatment costs. A value of £273 was assumed, based on NHS reference costs [6].

#### Human resource

Surveys were designed to generate primary research data to determine the time spent by Birmingham Midland Eye Centre doctors and nurses on managing MK patients. A nurse survey (Supplementary Table 1) established the time taken to administer single or multiple topical drugs. A doctors’ survey (Supplementary Table 2) determined the time spent on an initial patient consultation, investigations and admission.

Hourly rates for nurses and doctors were taken from the PSSRU costs [10]. It was assumed that patients would undergo a medical consultation at admission, including clinical assessments, prescribing and diagnostics, such as corneal scrapes. Any further medical consultation was assumed to take place during ward rounds, which form part of the bed-day cost. Further procedures did not require further medical staffing costs, as reference costs for procedures already had these incorporated [11]. It was assumed that every dose of topical medication was administered by a nurse; therefore, the total number of doses of topical medication (calculated as detailed below) was multiplied by the unit cost of a nurse’s time, in order to calculate the total cost of nurses administering topical drugs.

The unit cost of clinician time was calculated as the hourly rate from PSSRU, divided by the duration of patient contact.

#### Interventions

Costs of procedures (corneal biopsy, corneal glue, evisceration and temporary tarsorrhaphy) were estimated based on NHS Improvement figures, using the mean duration of theatre runtimes for specific procedures [11].

#### Investigations

E. *coli* overlay, blood, chocolate and Sabouraud agar plates, gram stains, CL testing and conjunctival swabs used on initial presentation were itemised on a per-patient basis, and the total cost estimated as per NHS reference costs [6]. PCR testing was conducted externally at a cost of £120 per patient, plus postage (£3.30) [12].

#### Drug costs

Drug costs were obtained from the British National Formulary drug prices [13].

1. Antimicrobial costs—these were itemised per patient and captured by the database. Each dose consisted of two drops, and the cost was calculated by multiplying the cost per drop by the total number of drops administered. The frequency of administration went through three stepdown treatment regimens for each patient, with the duration of each stage dependent on treatment response:
  Stage 1: administered hourly day and night (48 drops per day).
  Stage 2: administered hourly during the day, and two-hourly at night (36 drops per day).
  Stage 3: administered six times daily (12 drops per day)—at this stage, costs accounted for the size of the container and the shelf life of the treatment.
2. Anti-inflammatory steroid therapy: costs were calculated by multiplying the cost per dose by the frequency and duration.
3. Discharge medications: at discharge, patients were supplied with two weeks of discharge medications, the total costs of which were calculated.

#### Income

Hospital income was provided by the hospital coding department for each case.

### Statistical analyses

Prior to analysis, continuous variables were assessed using the Shapiro–Wilk test, and found not to be normally distributed. Hence, data are primarily reported as medians and interquartile ranges (IQRs) throughout, although mean costs are additionally reported, for ease of comparison with other studies. Demographic and treatment-related factors were compared between the disease severity grades using Mann–Whitney *U* tests for continuous variables, and Fisher’s exact tests for nominal variables. The calculated COAs were then compared to the income for each patient using Wilcoxon’s signed rank test. The association between the LOS and the COA to income discrepancy was then assessed using a regression model. Since this relationship was non-linear, the lengths of stay were log_10_-transformed, prior to analysis, in order to fit a logarithmic trend line to the data. The goodness of fit of this model was assessed by examination of the residuals, and the model was then evaluated to identify the point of cost neutrality, namely the LOS at which the estimated difference between COA and generated income was zero.

Analyses were performed using STATA^®^ (StataCorp. 2015) and SPSS Statistics for Windows, Version 25.0 (IBM Corp, Armonk NY). Statistical significance was defined as *p* < 0.05.

## Results

### Feasibility of data collection with the MOG form

The Excel based data collection form was tested on ten patients with two data collectors, and compared against the MOG form, to ensure all pertinent data were collected. With MOG being paper based, the fully electronic database added additional difficulties. One of these was attempting to minimise free text input, while allowing validated multiple selection inputs for single columns, particularly for risk factors and symptoms, antibiotic use and microbiology data, where patients may have multiple inputs under one variable. As this feature is not in-built in Microsoft Excel, data entry validation was performed using visual basic for application code. This allowed us to minimise the requirement of multiple columns for single variables, with multiple input options, allowing for a manageable electronic database (Supplementary File 2).

### Patient demographics

The *n* = 101 patients included in the analysis had a median age of 59 years (IQR: 38–73), and were predominantly of grade 4 disease severity (80.2%). Further details of demographics, clinical factors, risk factors, complications and surgical interventions during admission are reported in Table 1. Comparisons by disease severity found no significant differences between the grades 1–3 and grade 4 groups for any of the factors considered. No patients required corneal transplantation or amniotic membrane grafting.

**Table 1:** Patient demographics and clinical features by severity.

		Disease severity		
	Overall *n* = 101	Grades 1–3 (*n* = 20)	Grade 4 (*n* = 81)	*p* value
Age (years)	59 (38–73)	58 (42-73)	59 (35-74)	0.795
Gender (% male)	47 (47%)	9 (45%)	38 (47%)	1.000
Laterality (% right)	51 (50%)	12 (60%)	39 (48%)	0.445
Ethnicity (% white) [*n* = 99]	70 (71%)	15 (79%)	55 (69%)	0.576
More deprived^a^ (%)	70 (69%)	13 (65%)	57 (70%)	0.787
Time to presentation (days) [*n* = 94]	4 (2–7)	5 (3–7)	3 (2–7)	0.218
Length of stay (days)	7 (5-10)	7 (6-11)	7 (5–9)	0.575
Contact lens wear (any of the below)	26 (26%)	5 (25%)	21 (26%)	1.000
Soft fortnightly—soft monthly	13 (13%)	2 (10%)	11 (14%)	–
Daily disposables	2 (2%)	0 (0%)	2 (2%)	–
Rigid gas permeable	3 (3%)	1 (5%)	2 (2%)	–
Type not specified	8 (8%)	2 (10%)	6 (7%)	–
Other ocular risk factors (any of the below)	57 (56%)	12 (60%)	45 (56%)	0.804
Ocular surface disease^b^	36 (36%)	5 (25%)	31 (38%)	–
Foreign body	7 (7%)	1 (5%)	6 (7%)	–
Previous ocular surgery	23 (23%)	8 (40%)	15 (19%)	
Trauma	1 (1%)	0 (0%)	1 (1%)	–
Diabetes mellitus	8 (8%)	2 (10%)	6 (7%)	–
Topical immunosuppression	8 (8%)	1 (5%)	7 (9%)	–
Systemic immunosuppression	4 (4%)	0 (0%)	4 (5%)	–
Other	1 (1%)	0 (0%)	1 (1%)	–
Symptoms				–
Pain	72 (71%)	14 (70%)	58 (72%)	1.000
Red eye	67 (66%)	12 (60%)	55 (68%)	0.599
Photophobia	27 (27%)	8 (40%)	19 (23%)	0.162
Reduced Visual Acuity	22 (22%)	3 (15%)	19 (23%)	0.552
Blurred vision	21 (21%)	6 (30%)	15 (19%)	0.355
Epiphora	17 (17%)	5 (25%)	12 (15%)	0.319
Extra procedures (any of the below)	21 (21%)	2 (10%)	19 (23%)	0.232
Biopsy	0 (0%)	0 (0%)	0 (0%)	–
Evisceration	2 (2%)	1 (5%)	1 (1%)	–
Bandage lens	8 (8%)	0 (0%)	8 (10%)	–
Corneal glue	7 (7%)	0 (0%)	7 (9%)	–
Corneal debridement	2 (2%)	0 (0%)	2 (2%)	–
Temporary tarsorrhaphy	4 (4%)	0 (0%)	4 (5%)	–
Repeat scrape	4 (4%)	1 (5%)	3 (4%)	–
Botox	2 (2%)	0 (0%)	2 (2%)	–
Complications (any of the below)	17 (17%)	4 (20%)	13 (16%)	0.740
Endophthalmitis	1 (1%)	1 (5%)	0 (0%)	–
Phthisical	1 (1%)	0 (0%)	1 (1%)	–
Corneal perforation	6 (6%)	0 (0%)	6 (7%)	–
Re-admission	1 (1%)	1 (5%)	0 (0%)	–
Other	8 (8%)	2 (10%)	6 (7%)	–

Data are reported as median (interquartile range), with *p* values from Mann–Whitney *U* tests, or as *n* (%), with *p* values from Fisher’s exact tests.

^a^The proportion of patients in deciles 1–5 of the Index of Multiple Deprivation.

^b^Including atopy, dry eyes and cicatrising conjunctivitis.

### Direct costs analysis (COA) (*n* = 97)

Of the 101 patients, hospital coding records were only available in 97 (96.0%). The remaining four patients were not coded by the hospital, and so did not generate any income, hence were excluded from subsequent analysis. For the remainder, the COA was estimated for each patient, as previously described. For human resource costs, a survey of *n* = 30 doctors (Supplementary Table 2) estimated the mean consultation time to be 43.5 min (standard deviation [SD]: 14.0); the hourly rate was £140 [10], hence the unit cost per consultation was assumed to be £101.50. A survey of *n* = 4 nurses (Supplementary Table 1) estimated the mean time to administer topical medication (per drug, per eye) to be 4.1 min (SD: 1.7); the hourly rate was £41 [10], hence the unit cost per drug administration was assumed to be £2.80 per eye.

A summary of the total direct costs of care for patient admissions for MK is shown in Table 2. The median COA per patient was found to be £2855 (IQR: £2018–£4057), resulting in a total COA of £357,075 across the *n* = 97 patients (mean: £3681 per patient). The greatest contributors to this cost were the bed-day related costs (68.0%) and human resource costs (20.3%).

**Table 2:** Summary statistics for resource use per patient.

Category	Median cost (IQR)	Mean cost	% of total cost
Bed days	£1911.00 (£1365.00–£2457.00)	£2504.85	68.0%
Human resource	£644.70 (£426.30–£857.50)	£747.43	20.3%
Interventions	£0.00 (£0.00–£0.00)	£113.90	3.1%
Investigations	£35.00 (£28.00–£42.00)	£56.22	1.5%
Total drug costs	£243.78 (£147.68–£336.10)	£258.80	7.0%
Topical antimicrobials	£167.32 (£83.66–£236.77)	£185.90	5.1%
Systemic antimicrobials	£0.54 (£0.00–£11.20)	£3.68	0.1%
Topical glucocorticoids	£0.59 (£0.00–£2.36)	£1.68	0.0%
Medicines at discharge	£63.40 (£48.34–£92.62)	£67.54	1.8%
Total Cost	£2855.47 (£2018.38–£4057.02)	£3681.19	100.0%

Results are based on the *n* = 97 patients for whom hospital coding data were available.

*IQR* interquartile range.

### Income generated by the hospital

The total generated income through coding for the *n* = 97 admissions in 1 year was £252,116, with a median of £2124 per patient (mean: £2599). In total, seven different HRG codes were used, with the most common being “biopsy of lesion of cornea” (C511), which was used in *n* = 77 (79%) cases. The income and trim points varied widely across these codes, between 5 and 16 days and £891–£2,814, respectively, (Table 3). For *n* = 8 patients, the HRG code could not be identified, although an income was still generated in these cases. For the *n* = 4 patients that were excluded from the analysis due to lack of HRG coding, no income would have been generated. Based on the mean income per patient, not coding these cases could have represented an approximate loss of income of £10,396.

**Table 3:** Income and trim points of HRG codes.

HRG code	Description	Income	Trim point	*n*
C511	Biopsy of lesion of cornea	£2124	13	77
C474	Gluing of cornea	£2512	14	5
C202	Central protective suture of eyelid	£891	5	1
C013	Evisceration of the eye	£2814	16	3
C293	Occlusion of lacrimal punctum	£1204	5	1
C224	Injection into eyelid	£891	5	1
C515	Placement of therapeutic contact lens on to cornea	£2124	13	1
**–**	No specified code (1)^a^	£950	–	1
**–**	No specified code (2)^a^	£2106	–	7

Data are only reported for the *n* = 97 patients that received HRG codes.

^a^For *n* = 8 patients, no specific HRG code was recorded, although income was still generated in these cases.

#### Disparity between income generated and COA

**Fig. 1:**
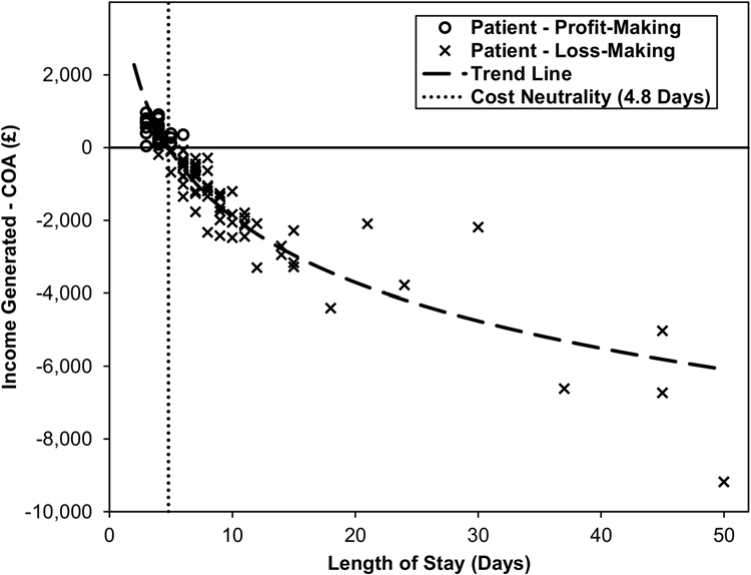
Association between income disparity and length of stay. The trend line is from a regression model, with Log_10_ length of stay as the independent variable and the difference between income generated and COA as the dependent variable. This found a significant association (*R*^2^ = 0.856, *p* < 0.001), with an estimated breakeven point (i.e. cost discrepancy = 0) of 4.8 days. Several coded income values were identical, hence some points are superimposed.

Income generated was found to be significantly lower than the COA (*p* < 0.001), with a median difference of £754 per patient (mean: £1082), yielding a total discrepancy of £104,960 across all *n* = 97 patients. Only 28 patients generated a “profit”, with the remainder (*n* = 69) being “loss-making”. Comparisons between these two groups found a significant difference in the average lengths of stay, with medians of 4 days (range: 3–6) and 8 days (range: 4–50), respectively, (*p* < 0.001). The association between LOS and the cost disparity was then further investigated using a regression model (Fig. 1). This found a significant logarithmic relationship (*R*^2^ = 0.856, *p* < 0.001), and estimated the breakeven point to be 4.8 days, above which patients were likely to be “loss-making”.

Four patients had total costs exceeding £10,000, and so were further interrogated. The most costly patient had a LOS of 50 days, as a result of social discharge difficulties. During this time, they accumulated a total cost of £20,651 against a generated income of only £11,470, with the main contributors to this cost being £13,650 in bed days and £5013 in human resource costs. The other three patients whose treatment costs exceeded £10,000 had lengths of stay ranging from 37–45 days, with total costs of £14,714–£16,763. The extended hospital stays were due to patients requiring evisceration (*n* = 1), corneal glue (*n* = 1) and social admission (*n* = 1).

#### Effect of indices of multiple deprivation and severity status

**Fig. 2:**
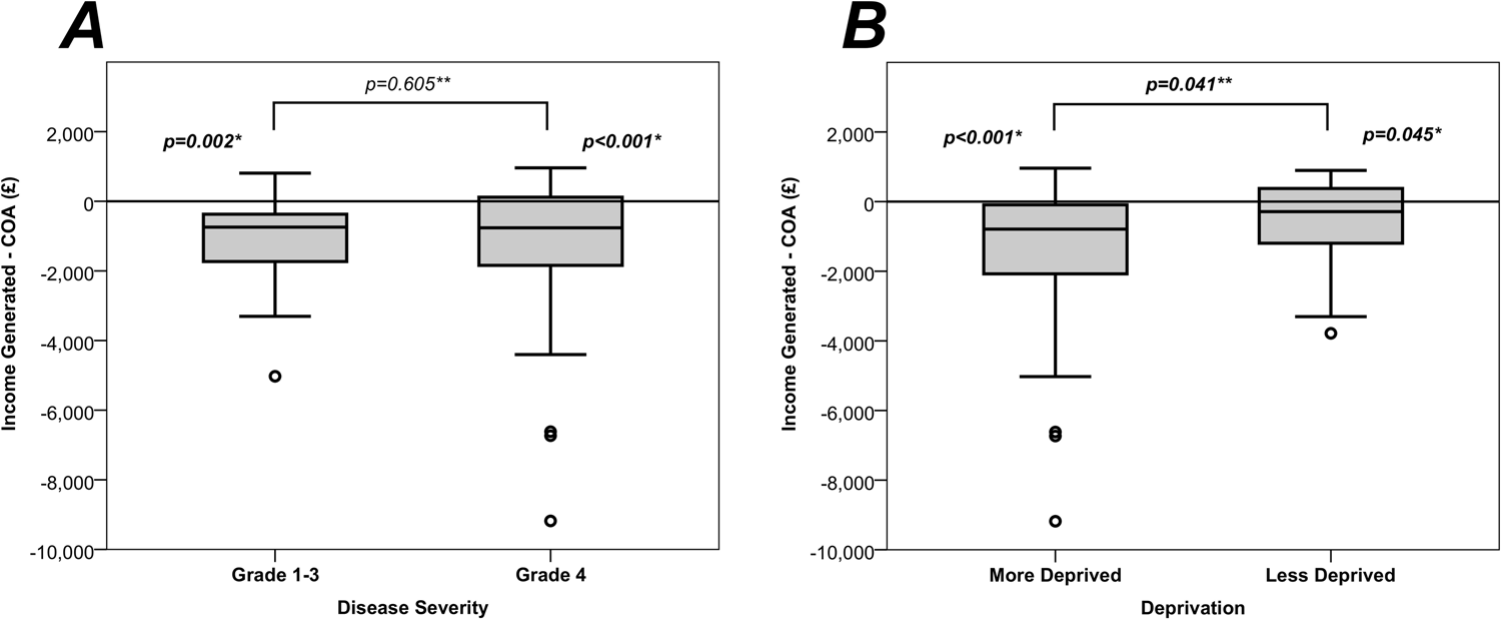
Association between income disparity and **a** disease severity and **b** deprivation. Deprivation was defined by the Index of Multiple Deprivation, with deciles 1–5 classed as “more deprived” and 6–10 as “less deprived”. *Within-group comparisons were performed using Wilcoxon’s signed rank tests between the income generated and direct cost of admission (COA). **Between-group comparisons were performed using Mann–Whitney *U* tests of the differences between income generated and COA. Bold *p* values are significant at *p* < 0.05.

This magnitude of discrepancy between income generated and COA was not found to differ significantly by disease severity, with median cost differences of £743 vs. £763 (mean: £1138 vs. £1068) for severity grades of 1–3 vs. 4 (*p* = 0.605, Fig. 2a). However, we found a significant difference when comparing the effects of socioeconomic deprivation, with the more deprived group having a greater cost discrepancy than the less deprived group, with median cost differences of £791 and £288 (mean: £1304 vs. £561) respectively (*p* = 0.041, Fig. 2b). To further investigate this finding, the COAs were compared between the two groups (Table 4), which found the more deprived cohort to have significantly higher human resource costs (*p* = 0.004), as well as higher total drug costs (*p* = 0.027), which were largely driven by increased costs of topical antimicrobials (*p* = 0.005). These differences were partly a result of the more deprived group tending to have longer durations of intensive day and night drops (median: 3 [IQR 2–4] vs. 3 [IQR 2–3] days, *p* = 0.058) and overall lengths of stay (median: 7 [IQR 5-10] vs. 6 [IQR 4–9] days, *p* = 0.126).

**Table 4:** Summary statistics for resource use by level of deprivation.

	More deprived	Less deprived	
Category	Median cost (IQR)	Median cost (IQR)	*p* value
Bed days	£1911.00 (£1365.00–£2730.00)	£1638.00 (£1092.00–£2184.00)	0.126
Human resource	£704.90 (£483.70–£930.30)	£493.50 (£389.90–£672.70)	**0.004**
Interventions	£0.00 (£0.00–£0.00)	£0.00 (£0.00–£0.00)	0.652
Investigations	£35.00 (£28.00–£144.50)	£28.00 (£28.00–£35.00)	0.336
Total drug costs	£253.74 (£166.90–£365.93)	£199.32 (£81.26–£280.22)	**0.027**
Topical antimicrobials	£187.32 (£96.11–£268.25)	£118.98 (£39.81–185.24)	**0.005**
Systemic antimicrobials	£0.80 (£0.00–£11.20)	£0.00 (£0.00–£3.66)	0.117
Topical glucocorticoids	£0.59 (£0.00–£2.36)	£0.59 (£0.00–£2.36)	0.938
Medicines at discharge	£61.23 (£49.43–£92.14)	£79.32 (£8.30–£92.62)	0.614
Total cost	£2900.92 (£2207.36–£4250.86)	£2394.51 (£1747.56–£3319.14)	0.062

Results are based on the *n* = 97 patients for whom hospital coding data were available. Data are reported as median (interquartile range), with *p* values from Mann–Whitney *U* tests. Bold *p* values are significant at *p* < 0.05. Deprivation was defined by the Index of Multiple Deprivation, with deciles 1–5 classed as “more deprived” and 6–10 as “less deprived”.

## Discussion

We have demonstrated that the MOG-derived data collection toolset (Supplementary File 2) captures pertinent clinical data for quantification of direct costs of MK admissions in the UK. In our retrospective test cohort, there was a significant financial deficit, particularly with longer admission, suggesting that available codes do not reflect the true cost of treating MK within the NHS, and that a specific treatment tariff should be established. In addition, as a variety of HRG codes are used without consistency, similar patients may generate significantly different income. Through extrapolating our data by the number of MK admissions found on 2014/15 NHS Digital data (*n* = 2617), the estimated financial deficit for NHSE amounts to £2,831,745 per annum.

Health-economic analyses showed that LOS was the key driver for direct costs of care for patients, with the pivotal LOS of 4.8 days indicating cost neutrality. Based upon our test cohort, the patient care pathway should encourage discharge of patients capable to self-administer treatment after the sterilisation phase to enable efficiency and financial parity. Low socioeconomic status has been associated with increased incidence of blindness and visual impairment [14, 15], and we have demonstrated a significantly higher cost in those patients with higher deprivation scores.

Few studies evaluate MK clinical outcomes prospectively [1, 16–18]. Although these studies report the clinical outcomes in terms of visual acuity and complications, none determine the economic impacts. Keay et al.’s description of a CL-related MK grading attempted to relate severity scoring to disease burden in the form of direct costs (medical care, pathology and medications) and indirect costs (loss of income, assistance of carers and purchase of spectacles) in a group of 278 patients. These data demonstrated a statistically significant inverse relationship between disease duration and severity, a comparable trend to our data, but with shorter resolution time. This difference is likely to be attributed to our inpatient cohort and varying definition of disease resolution in the studies.

The mean COA per patient was £3681, compared to a mean income of £2599, resulting in a significant shortfall of £1082 per patient. Direct costs were comparable to the Australian data (£0.45 to Australian $1 currency conversation rate estimated at the time of publication in 2008, to aid comparison with our data), which reported costs of Australian $5515 (IQR $2784–$9437)/£2482 (IQR £1253–£4247) for severe cases with vision loss, Australian $1596 (IQR $774–$4888)/£3547 (IQR £348–£2200) for severe cases without vision loss and Australian $795 (IQR $527–$1234)/£358 (IQR £237–£555) for mild MK (ANOVA, *p* = 0.001) [8]. Unlike the Australian study, our data did not demonstrate a significant difference in cost for admissions of presumed MK by disease severity using the Keay criteria. As these criteria were developed for grading of contact lens associated keratitis, our findings suggest that this grading system may be appropriate for guiding management decisions in the community or by optometrists, but might not be the most suitable system to inform clinical and economic decision making at the tertiary care level. As such, an alternative disease severity grading system should be developed and validated for stratifying MK admissions in regional centres. Future studies involving a nationwide dataset to increase the statistical power of the analyses would help tease apart whether other factors impact upon COA, such as: (1) early identification of bacterial pathogen, (2) the presence of a bacterial pathogen, (3) gram staining of the organisms, (4) the presence of atypical pathogens (fungi, amoeba, mycobacteria, nocardia, microsporidia) and (5) a broader reach of the IMD data to analyse each decile.

Collier et al. estimated the total keratitis costs in the USA in 2010 to be $174.9 million, although this has several key limitations. Firstly, they include reporting of all keratitis, rather than just infectious cases. In addition, they utilised insurance claims data for their data capture methodology, which would inherently exclude optometric care of MK, as this is not typically covered by health insurance in the USA. This leads to the exclusion of milder keratitis cases that present to, and are treated by optometrists in the USA [19].

Estimates of international treatment costs vary widely in existing studies. The mean treatment cost in this study is higher than any other identified study, including those conducted from a societal perspective. This may be due to the majority of our cohort having high disease severity, which Keay et al. [8]. found to be associated with higher treatment costs, although no such effect was observed in our study.

Identifying a microbial pathogen for every case of clinically presumed MK is not possible due to sensitivity and specificity issues relating to diagnostic techniques currently available to clinicians to detect causative organisms in presumed MK. Corneal scrapes have a widely varying sensitivity rate in the literature (23.7–61.5%) [3]. The management of patients who are pathogen-negative are likely to be false-negative, and these patients should not have management strategies eased due to the absence of a detected organism if the clinical features are in keeping with MK. Detection of a microbial pathogen should therefore not be a prerequisite to the process of establishing a new HRG code for patients treated as hospital admissions with presumed MK.

In summary, our study is the first to attempt to calculate the cost burden of MK admission amongst NHS patients in the UK and identify the need for appropriate coding for treatment of MK in a hospitalised setting. We have confirmed that the MOG-derived data collection toolset captures pertinent clinical data that allow quantification of direct costs of patient care for MK. However, further development of this toolset is required to include measures that capture indirect costs of disease burden, including the impact of treatment (particularly in those who self-treat at home) and visual morbidity on patients’ quality of life. In addition, since socioeconomic deprivation was found to be associated with both LOS and treatment costs, collection of residential postcodes would also be warranted, to assess whether aetiological pathogens, severity of presentation and the disease course vary with social deprivation, or between rural and urban domiciles. The process of transposing data fields into a multiuser database platform with multisite access is currently underway, to enable a national patient registry to be established, data from which could be used to validate the current findings in a larger prospective patient cohort regionally and/or nationally, and ultimately gather insight into the economic burden of MK in the UK and elsewhere.

### Summary

#### What was known before

- MK is the most common non-surgical ophthalmic emergency hospital admission in the UK.
- There is no specific HRG code to standardise income for treatment of this common pathology.

#### What this study adds

- Quantification of the significant direct-cost burden of MK admissions on the NHS.
- Development of a MOG-derived toolset for curating health-economic data and direct costs of care.
- Greater socioeconomic deprivation is demonstrated to carry significantly increased costs of care per hospital admission for MK.

## Supplementary information

Supplementary File 1

Supplementary Tables

Supplementary Figure 1

Supplementary File 2
