# Supplementary material for: Calculating the economic burden of presumed microbial keratitis admissions at a tertiary referral centre in the UK
Source: Eye (Lond). 2020 Dec 7;35(8):2146–54. doi: 10.1038/s41433-020-01333-9 (PMC8302743; doi:10.1038/s41433-020-01333-9)
Supplement: Supplementary file 1 [file 41433_2020_1333_MOESM1_ESM.docx]

**Microbiology Ophthalmic Group (Clinical Proforme)**

**^M^Centre …………………… ^M^Laboratory reference number …………….**

**^M^Hospital …….**

Consultant……

**^M^Patient details (attach label)**

Surname ……………….

First Name ………………

Hospital Number………

Date of birth …………....

***Relevant history****:*

***Relevant systemic diagnosis/es*** *……………………………*

*……………………………………………………………..*

***Relevant systemic medication*** *…………………………….*

*……………………………………………………………...*

###### *Topical Antimicrobial/s used within last 14 days*

#### None / Chlor / Gent/ Amitk /Ceftaz / Cefurox / Cipro/

#### Fuc / Teico/ Oflox / Pen / Vanc / Pen / Unknown /

*Other*…………………………………………….

Still using at presentaion Yes / No

*Other Ocular medication within last 14 days*

*Acyclovir / steroid / other ………………….*

*Still using at presentation Yes / No*

| ^M^Aetiology (Must be completed) | | | | | | |
| --- | --- | --- | --- | --- | --- | --- |
|  | **No** | **Yes** | Type | | | |
| **Contact Lens** |  |  | GPC | Soft  Daily | Daily Disp | Soft  Extend |
| **FB** |  |  | Metallic | Organic | Suture | Other |
| **Previous Keratitis** |  |  | HSV | Fungal | Bacterial | Acanth |
| **Corneal Disease** |  |  | Dyst | Surgery | Transplt | Other |
| **External Disease** |  |  | Ant. Lid | Post lid | Trichiasis | Acne R |
|  |  |  | Other OSD | Toxic | Immune OSD | Atopy |
| **Corneal Trauma** |  |  | Weapon | Chemical | Thermal | Animal |

## Outcome

**^M^Re-epithelialised (Date)…. ……………..^M^Days ……….**

# ^M^Antimicrobial change? N / Y=…………………………

**^M^Days on treatment ………..….** *Compliance: Good / Poor*

*Visual Acuity ………………*

### Presentation

**^M^Presentation (Date)…………………..…….**

**^M^Treatment prescribed:……………………..**

**…………………………………………...**

*Visual acuity*……………..…………………………

#### Draw

*Draw*

###### *Scar* : *Yes/No*

*Major axis …………..*

*Minor axis ………….*

*Shortest distance to limbus ……….*

*Thinning a) None b) 1-25% c) 50-75%*

*Vascularisation (Draw) Quads: Sup..…..Deep….….*

###### *Ulcer Details*

*Major Axis ………………………..*

*Minor Axis ………………………..*

*Shortest limbal distance …………..*

*Thinning a) 1-25% b) 26-50% c) 51-75%*

*d) >75% e) perforation*

**^M^Corneal scrape: Date ……………………**

**^M^Repeat Scrape Yes /No Date …………………….**
