# Supplementary material for: Calculating the economic burden of presumed microbial keratitis admissions at a tertiary referral centre in the UK
Source: Eye (Lond). 2020 Dec 7;35(8):2146–54. doi: 10.1038/s41433-020-01333-9 (PMC8302743; doi:10.1038/s41433-020-01333-9)
Supplement: Supplementary file 2 — Supplementary Tables [file 41433_2020_1333_MOESM2_ESM.docx]

Supplementary Table 1: Nurse Survey Results

| **Number of**  **medicine administrations** | **Time (Minutes)** | | | | | **Mean per**  **Treatment** |
| --- | --- | --- | --- | --- | --- | --- |
|  | **Nurse 1** | **Nurse 2** | **Nurse 3** | **Nurse 4** | **Mean** |  |
| 1 administration | 1 | 6 | 1 | 6 | 3.5 | 3.5 |
| 2 administrations | 5 | 11 | 5 | 12 | 8.25 | 4.1 |
| 3 administrations | 15 | 13 | 15 | 15 | 14.5 | 4.8 |
| 4 administrations | 15 | 15 | - | 16 | 15.3 | 3.8 |
| **Overall Mean Time per Treatment (Minutes)** | | | | | | **4.1** |

Supplementary Table 2 - Doctor Survey Results

| **Initial Consultation (Minutes)** | **Additional Time (Minutes)*** | **Total Consultation**  **Time (Minutes)** |
| --- | --- | --- |
| 45 | - | 45 |
| 60 | 10 | 70 |
| 45 | 10 | 55 |
| 60 | 10 | 70 |
| 60 | - | 60 |
| 60 | - | 60 |
| 60 | - | 60 |
| 30 | - | 30 |
| 50 | - | 50 |
| 50 | - | 50 |
| 40 | 10 | 50 |
| 20 | - | 20 |
| 30 | - | 30 |
| 30 | 15 | 45 |
| 30 | - | 30 |
| 20 | 20 | 40 |
| 45 | 10 | 55 |
| 30 | 5 | 35 |
| 40 | - | 40 |
| 30 | 5 | 35 |
| 15 | 5 | 20 |
| 45 | 10 | 55 |
| 20 | - | 20 |
| 25 | 5 | 30 |
| 30 | - | 30 |
| 45 | 10 | 55 |
| 40 | - | 40 |
| 30 | - | 30 |
| 50 | - | 50 |
| 45 | - | 45 |
| **Overall Mean Time per Consultation (Minutes)** | | **43.5** |

*If clinicians specified a time for ’additional contact’ they were asked to provide detail. Responses included referrals, imaging, clerking, counselling patients and testing. This time was therefore assumed to form part of the overall time spent on the initial patient consultation. Thus, if clinicians specified additional contact time, the time was added to their specified ‘initial consultation’ time and mean duration of a consultation was calculated using the sum of both times.
