# Supplementary material for: Calculating the economic burden of presumed microbial keratitis admissions at a tertiary referral centre in the UK
Source: Eye (Lond). 2020 Dec 7;35(8):2146–54. doi: 10.1038/s41433-020-01333-9 (PMC8302743; doi:10.1038/s41433-020-01333-9)
Supplement: Supplementary file 3 — Supplementary Figure 1 [file 41433_2020_1333_MOESM3_ESM.pdf]

**Supplementary Figure 1**

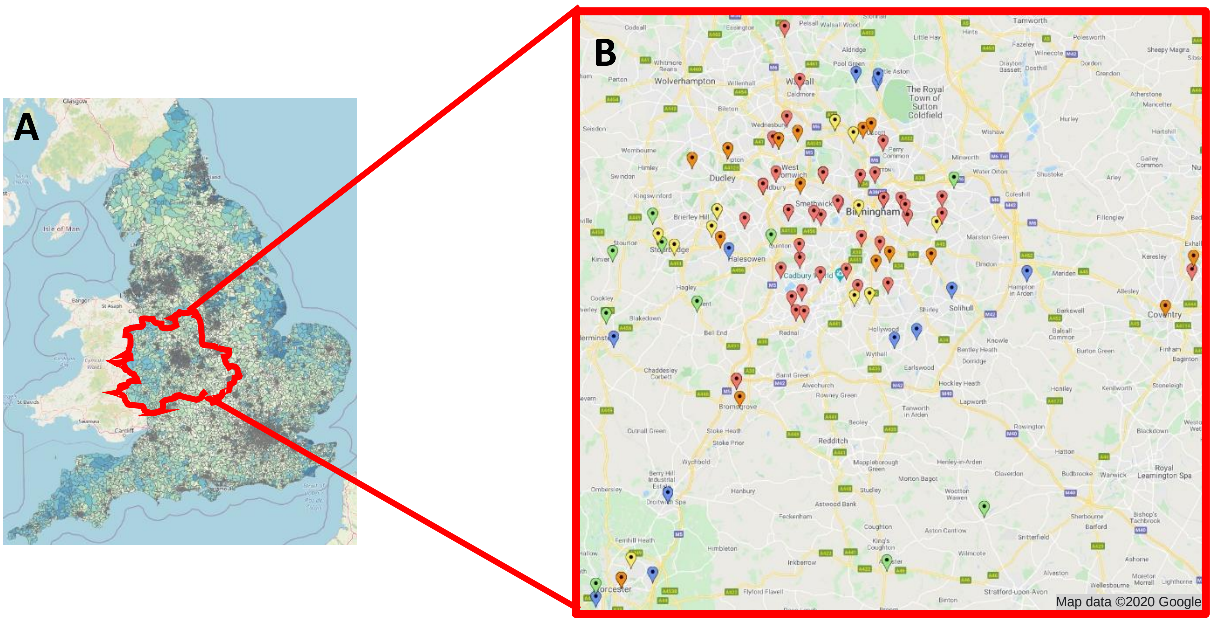


**A**) Indices of Deprivation Rank Map from Gov.uk 2019

**B**) Location of patients from grouped by IMD Deciles.

| | |
|---|---|
| | Most Deprived (IMD Deciles 1-2) |
| | IMD Deciles 3-4 |
| | IMD Deciles 5-6 |
| | IMD Deciles 7-8 |
| | Most Affluent (IMD Deciles 9-10) |
